# The Immunogenicity of Peptoid-Protein Conjugates

**DOI:** 10.4172/2157-7560.1000329

**Published:** 2016-07-11

**Authors:** Allison Case, Angela Desmond, Daniel Lopes, Kelly Dye, Kelly Mapes, Stephen Ruback, Iliodora Pop, Jiyeun Kate Kim, Pavitra Chakravarty, Joan E. Smallshaw, Laurentiu M. Pop, Ellen S. Vitetta

**Affiliations:** 1 Cancer Immunobiology Center and Department of Immunology, University of Texas Southwestern Medical Center, Dallas, Texas, USA; 2 Department of Bioengineering, The University of Texas at Dallas, Texas, USA; 3 Department of Pediatrics, Feinberg School of Medicine, Northwestern University, Chicago, Illinois, USA; 4 Eli Lilly, Indianapolis, Indiana, USA; 5 School of Medicine, Texas Tech University Health Sciences Center, Lubbock, Texas, USA; 6 Green Center for Systems Biology, University of Texas Southwestern Medical Center, Dallas, Texas, USA; 7 Department of Neuroscience, University of Texas Southwestern Medical Center, Dallas, Texas, USA; 8 College of Pharmacy, Pusan National University, Busan, Republic of Korea; 9 Department of Internal Medicine, University of Texas Southwestern Medical Center, Dallas, Texas, USA; 10 Department of Microbiology, University of Texas Southwestern Medical Center, Dallas, Texas, USA

## Abstract

We demonstrate that a peptoid composed of five monomers and attached *via* a maleimide linker to a carrier protein elicits anti-peptoid, anti-linker and anti-carrier antibodies in rabbits. Specific anti-peptoid antibodies were affinity purified and used to reproducibly retrieve three specific peptoid-coupled beads from 20,000 irrelevant peptoid-beads using magnetic screening.

Our long term goal is to generate vaccine candidates for any pathogen or toxin for which neutralizing polyclonal antibodies (PAbs) or monoclonal antibodies (MAbs) exist or could be made, without prior knowledge of the protective epitope(s). To this end, we are developing a platform to screen large libraries of B cell epitopes with known neutralizing antibodies. For our synthetic B cell epitopes, we are using peptoids [1], which are polymer chains of N-substituted glycines. Peptoids are similar to peptides (Figure 1) [2], but might have several advantages over peptides with regard to vaccines. First, peptoids are protease-resistant haptens that will not elicit and antibody response unless they are attached to a carrier protein [3]. Although the production of high affinity neutralizing IgG antibodies requires interactions between hapten (peptoid)-specific B cells and carrier specific T cells, even when attached to a carrier protein, peptoids should not be degraded in the blood or tissues. Therefore, unlike the carrier protein, which must be degraded to activate T cells, the protease resistance of the peptoid (as compared to a peptide) is advantageous. For human use, peptoids mimicking epitopes from one or more pathogens or toxins could be attached to an approved protein vaccine such as tetanus toxoid. Second, since the structure of the peptoid is unrelated to the structure of the native B cell epitope, except for its “shape”, it should be possible to select peptoids that fit the binding site of any broadly neutralizing screening antibody, regardless of whether that antibody recognizes a linear or conformational epitope. Finally, as compared to peptides which are restricted to amino acids, peptoids have virtually unlimited diversity based on the R groups coupled to the nitrogens.

When a peptoid is identified in this new vaccine platform, it is sequenced, synthesized and coupled to a protein using a synthetic linker. In a previous study, a pool of peptoids did not elicit antibody responses in mice unless they were conjugated to a protein (3). However, the contribution of anti-peptoid, anti-linker and anti-carrier protein antibodies to the total antibody response was not studied.

To fully characterize the antibody response against peptoid-protein conjugates and to determine whether anti-peptoid antibodies can recognize both carrier-conjugated and bead-coupled peptoids, two peptoids were synthesized as described previously [4]. One was a five monomer immunizing peptoid (R5). The other was a seven monomer control peptoid (RC). They were each linked via a maleimide linker (Pierce, Rockford, IL) to a carrier protein as shown in Figure 2. The monomers used to synthesize the peptoids were chosen for their chemical diversity (e.g., aliphatic, aromatic, charged) and to maintain a molecular weight (MW) difference of at least one Dalton so the identity of each monomer could be distinguished by matrix assisted laser desorption/ionization tandem mass spectrometry (MALDI-TOF MS/MS) (Applied Biosystems, Carlsbad, CA). Keyhole limpet hemocyanin (KLH; Pierce) was the carrier protein used for conjugation and immunization. R5-KLH conjugates were emulsified in the adjuvant, aluminum hydroxide (alum) (Alhydrogel; Accurate Chemical and Scientific Corporation, Westbury, NY) and used to immunize two New Zealand rabbits. The rabbits were bled prior to immunization and exsanguinated after several boosters. To quantify the levels of anti-KLH, anti-linker and anti-R5 peptoid antibodies (anti-R5; RAR5) in the sera and to confirm their specificity before and after affinity purification, enzyme linked immunosorbent assays (ELISAs) were performed. Ninety six-well ELISA plates (BD Bioscience, San Jose, CA) were coated with either R5 peptoid conjugated to bovine serum albumin (BSA; Pierce) (an irrelevant carrier protein) (R5-BSA), BSA alone, KLH alone, the immunizing conjugate (R5-KLH), an irrelevant peptoid conjugated to BSA (RC-BSA), or ovalbumin (OVA) alone. After washing and blocking, dilutions of pre-immunization sera, pre-affinity purified antisera and post-affinity purified antibodies were added and incubated. After washing, horse radish peroxidase (HRP)-labeled goat anti-rabbit IgG secondary antibodies (Jackson Immuno Research, West Grove, PA) were added. The plates were developed by adding a 3, 3′, 5, 5′-tetramethylbenzidine (TMB; Pierce) substrate. Absorbance was measured with a plate reader (Molecular Devices, Menlo Park, CA, USA) at λ=450 nm.

No antibodies against peptoid, linker or carrier protein were present in the pre-immunization sera (data not shown). Following immunization and boosting, antibodies against the intact peptoid, linker and carrier protein could be detected (Figure 3A). After absorption with sepharose (Sigma Aldrich) coupled to KLH and affinity purification on sepharose coupled to R5 via an irrelevant linker (SulfoLink Immobilization Kit for Peptides, Pierce) the affinity purified PAbs recognized only the R5 peptoid (Figure 3B). These antibodies did not react with the control RC peptoid (Figure 3A). Proteins were separated by 4–15% sodium dodecyl sulfate polyacrylamide gel electrophoresis (SDS-PAGE) (GE Healthcare, Piscataway, NJ) under non-reducing and reducing conditions using a PhastGel System (GE Healthcare). The gel was stained with PhastGel Blue R (GE Healthcare). The MW of the affinity purified rabbit anti-R5 (RAR5) antibodies was consistent with that of IgG molecules (data not shown).

We further confirmed the presence of anti-linker antibodies in the antiserum but not in the affinity purified RAR5 by attaching an irrelevant carrier (BSA; Pierce) to the maleimide linker and testing the sera and affinity purified PAbs against ovalbumin or ovalbumin-maleimide by ELISA (Figure 4). As shown in Figure 4A, RAR5 reacted with the linker. However by absorbing the anti-KLH antibodies and affinity purifying the RAR5, only anti-R5 peptoid antibodies remained (Figure 4B). These affinity purified RAR5 PAbs were then used to retrieve three R5 peptoid-coupled TentaGel beads (Rapp Polymere; Tubingen, Germany) “spiked” into 20,000 irrelevant RC-coupled peptoid TentaGel beads using a magnetic screening assay. For each round of screening, beads retained by the magnet were counted using a light microscope at 40× power. The sequence of the peptoid from the recovered beads was determined after cleaving the peptoid from the bead using a cyanogen bromide (CNBr) solution, and analyzing the peptoid sequence by MALDI-TOF MS/MS. Beads not retained by the magnet were removed by pipetting, washed, and again incubated with two increasingly higher concentrations of RAR5. As shown in Table 1, when three R5 peptoid beads were spiked into ~20,000 RC peptoid beads, the former could be retrieved reproducibly at a concentration of 0.1 µg/ml RAR5.

Our findings show that when conjugated to carrier proteins, antibodies against the peptoid, linker and carrier protein are made. The purified anti-peptoid antibodies were free of anti-linker and anti-carrier protein antibodies and did not react with a control peptoid. Using this affinity purified RAR5 PAbs in a conventional magnetic screening assay, we demonstrated that they also recognized the R5 peptoids displayed on TentaGel beads. Thus, antibodies made against the peptoid conjugated to a carrier elicited anti-peptoid antibodies that could also recognize peptoids attached to beads. This strongly suggests that some or all of the immunogenic peptoid epitopes are preserved whether or not they are attached to beads.

Based on these results concerning immunogenicity, we are screening large peptoid libraries with well-defined purified PAbs or MAbs. Future studies will be designed to confirm preliminary findings regarding the diversity of peptoid libraries necessary to identify mimetics of the complex B cell epitopes recognized by broadly neutralizing MAbs. We will then determine whether peptoid-carriers induce cross-reactive mimetic antibodies that can neutralize pathogens or toxins.

## Figures and Tables

**Figure 1:**
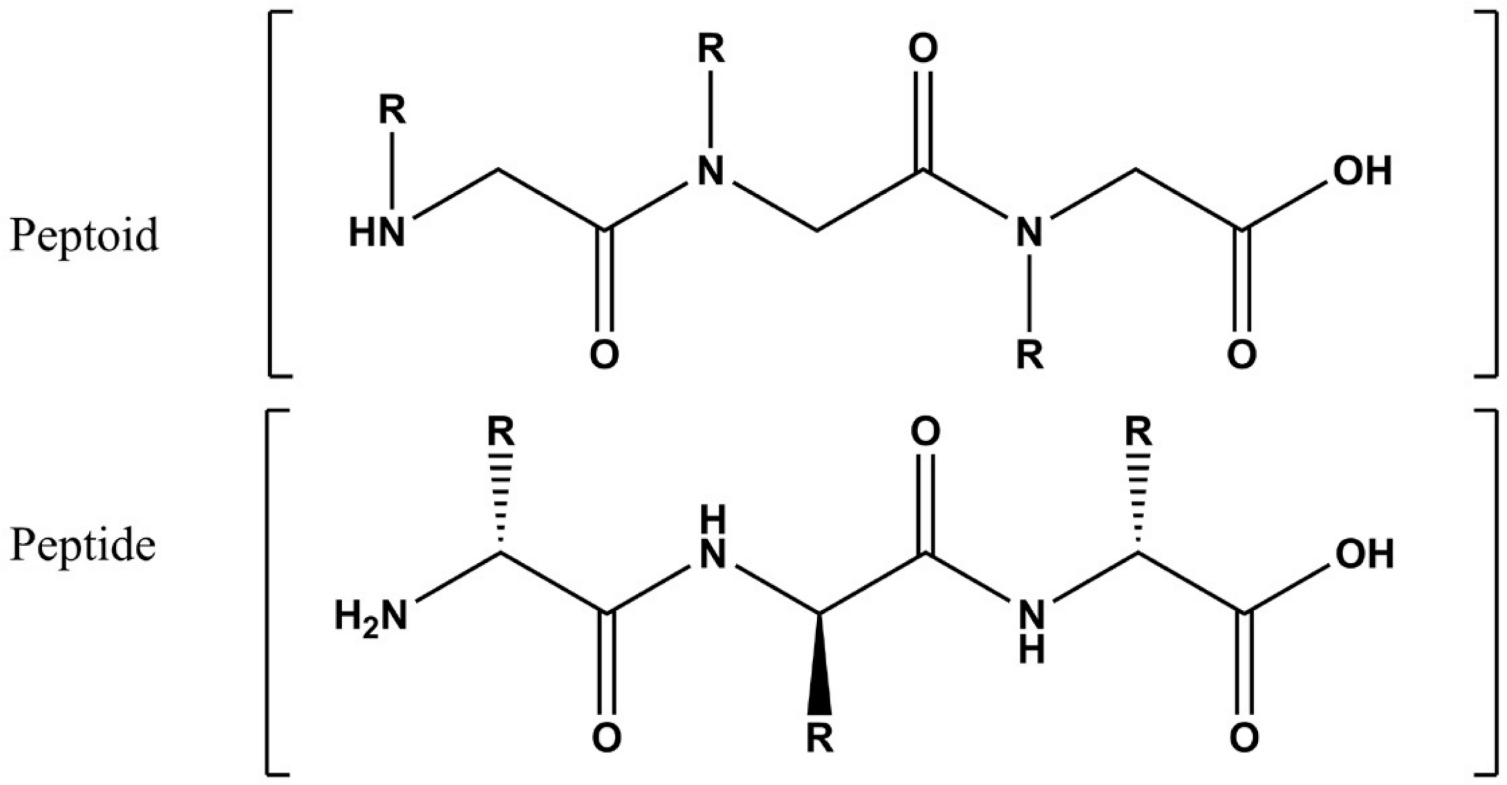
Structure of peptoids versus peptides. R symbolizes a side group [1].

**Figure 2:**
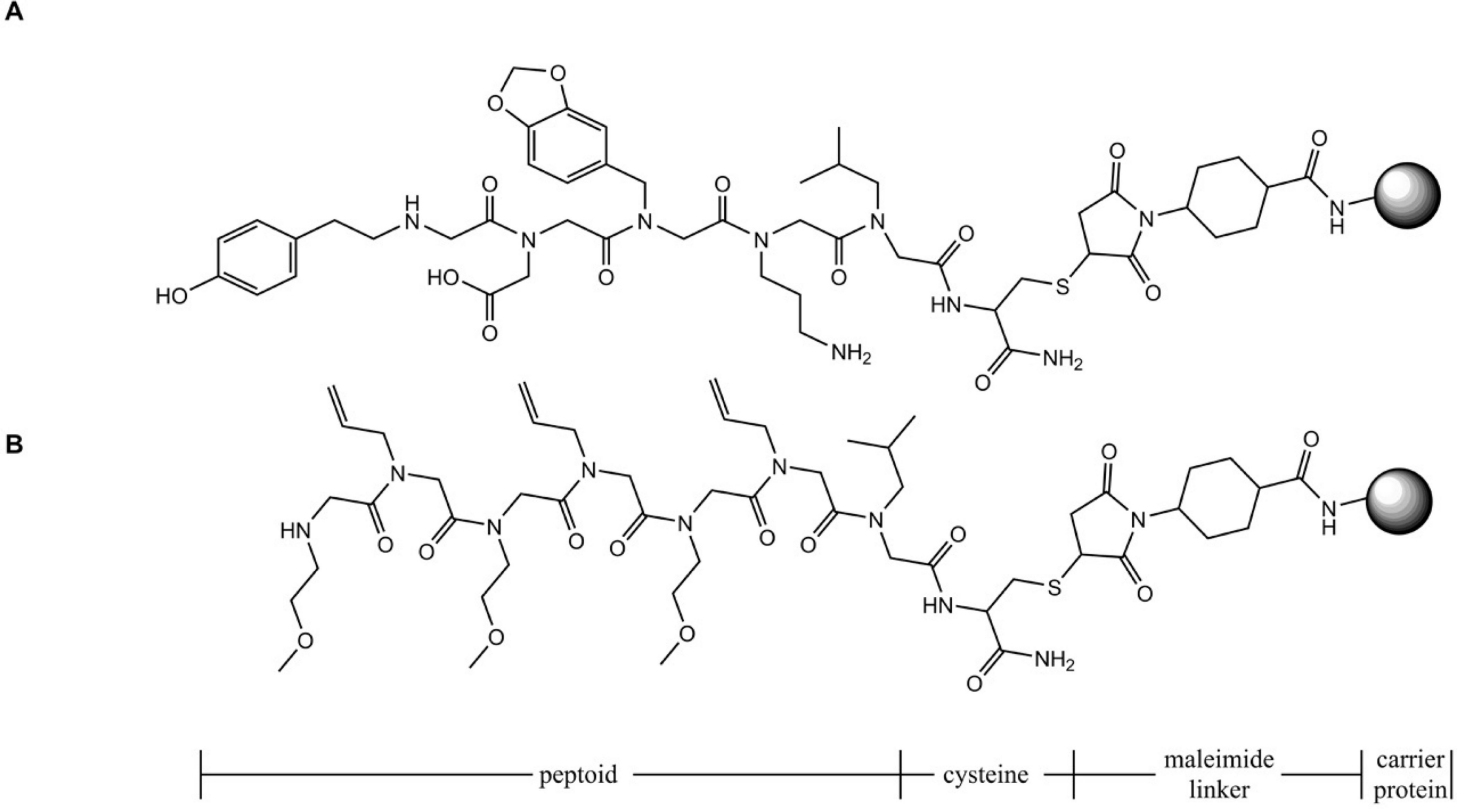
Schematic representation of R5 and RC peptoids conjugated to maleimide-activated carrier proteins. **A)** R5 peptoid was synthesized with a C-terminal cysteine (Cys) residue to allow conjugation to the maleimide-activated carrier protein. The R5-KLH conjugate was used to immunize rabbits. **B)** RC peptoid was synthesized with a Cys as described for R5 and used as an irrelevant control for R5 peptoid. Note that RC shares the first peptoid monomer with R5 to aid in the detection of anti-linker antibodies.

**Figure 3:**
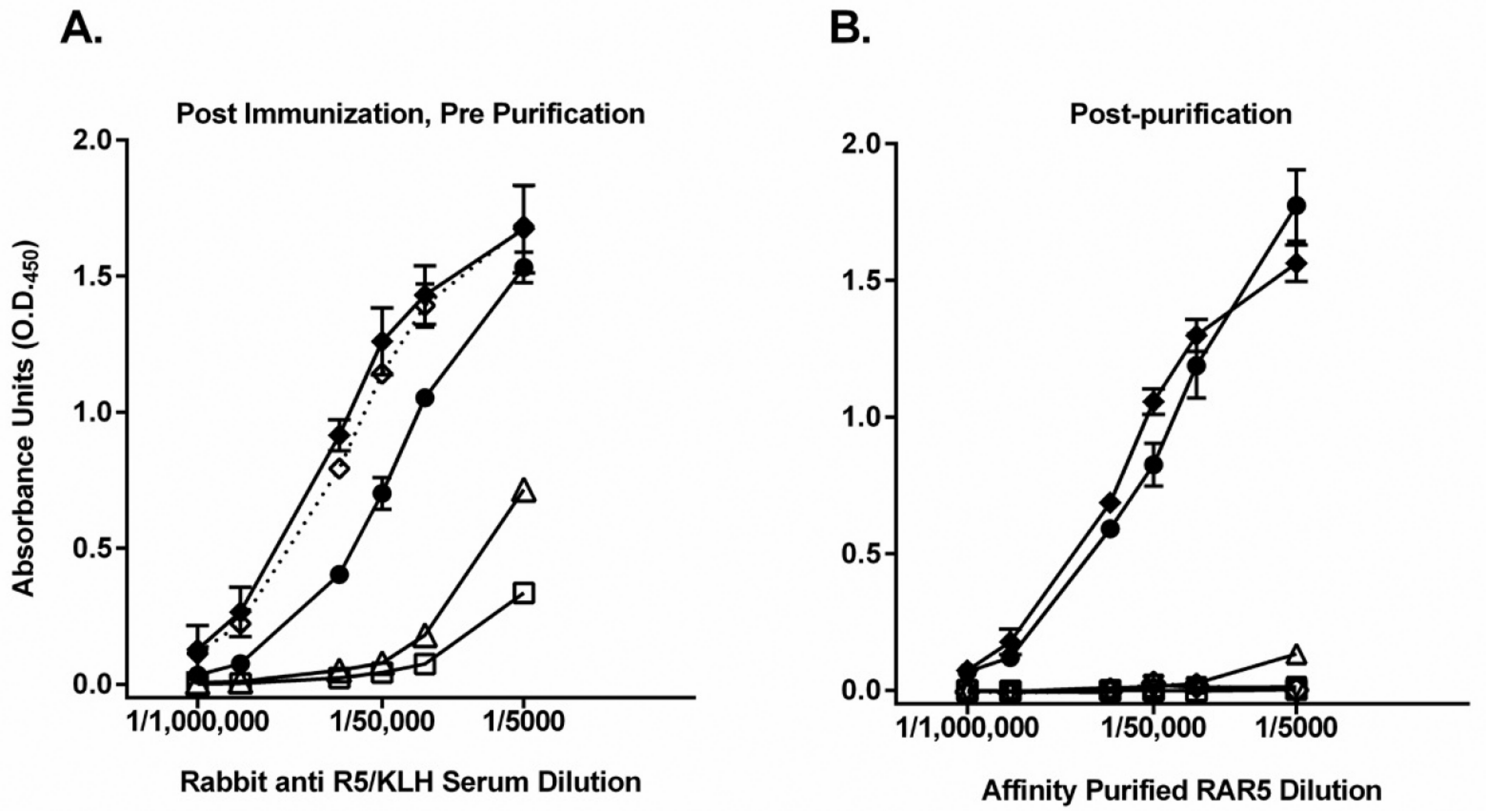
Immunization with R5- maleimide-KLH generated specific anti-R5 peptoid antibodies. A. Analysis of rabbit sera after immunization with R5-KLH by ELISA demonstrated the specificity of the RAR5 antibodies. ELISA plates were coated with R5 peptoid conjugated to an irrelevant carrier protein (R5-BSA,•); an irrelevant peptoid conjugated to BSA (RC-BSA, Δ) using the same linker as was used to link R5 to KLH used for immunization; BSA alone (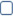); KLH alone (◊ with dotted line); and R5-KLH (♦). Dilutions of pooled, RAR5 serum, before affinity chromatography, from two rabbits were then added and detected using a species-specific enzyme-conjugated secondary antibody and an appropriate substrate. B. Dilutions of pooled rabbit anti serum from two rabbits was purified and the ELISA repeated. ELISA plates were coated with R5 peptoid conjugated to an irrelevant carrier protein (R5-BSA, •); an irrelevant peptoid conjugated to BSA (RC-BSA,Δ) using the same linker as was used to link R5 to KLH used for immunization; BSA alone (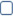); KLH alone (◊ with dotted line); and R5-KLH (♦). Data represent one of three experiments, and are presented as the average +/− standard deviation of triplicate wells. Data were normalized to baseline (the absorbance detected with no primary antibody. A one tailed, paired student’s t test was performed (p<0.01) (A and B).

**Figure 4:**
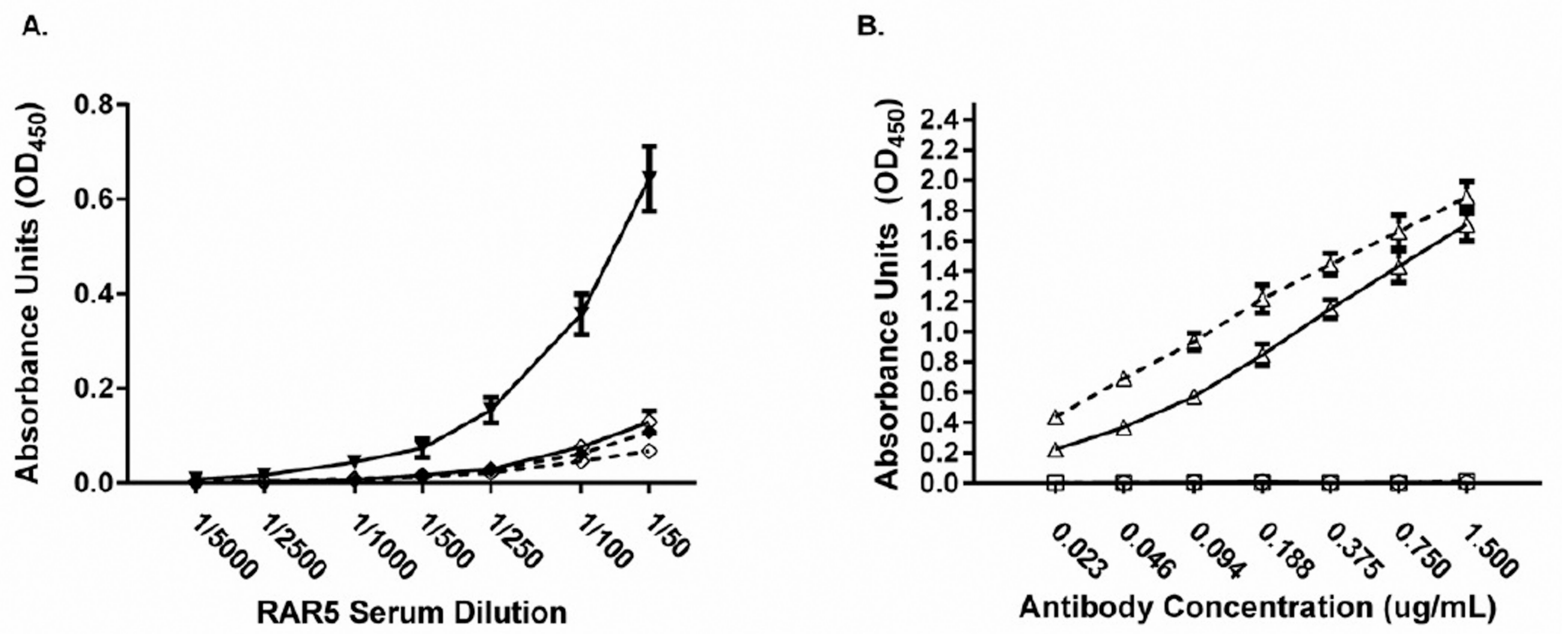
Immunization of a rabbit with R5-maleimide-KLH generated specific polyclonal anti-maleimide antibodies as shown by ELISA. **A)**. ELISA plates were coated with ovalbumin (dotted line) or ovalbumin-maleimide (solid line) and serial dilutions of either pre-bleed serum (◊) or pooled, post-immunization serum (♦) were added. **B)**. ELISA plates were coated with ovalbumin (dotted line) or ovalbumin-maleimide (solid line) and serial dilutions of purified, polyclonal rabbit anti-R5 peptoid antibodies (○), rabbit anti-ovalbumin (Δ), or normal rabbit IgG (▢) were added. Both ELISAs were performed using a species-specific enzyme-conjugated secondary antibody and its corresponding substrate. Data represent triplicate assays and are presented as the average of triplicate assays +/− standard deviation. Data were normalized to baseline (the absorbance detected with no primary antibody. A one tailed paired student’s t test was performed for post immunization serum (p<.01) (left panel) and for post purification serum (p<0.01) (right panel).

**Table 1:** The retention by magnetic screening of three R5 peptoid beads added to pools of ~20,000 irrelevant RC peptoid beads is highly sensitive and specific.

RAR5 concentration (µg/mL)	Beads retained by magnetic screening			MALDI-confirmed R5 peptoid beads			MALDI-confirmed RC peptoid beads			Beads unreadable by MALDI			Sensitivity* (%)		
Experiment:	1	2	3	1	2	3	1	2	3	1	2	3	1	2	3
0	0	1	0	-	0	-	-	1	-	-	0	-	-	0	-
0.01	0	0	0	-	-	-	-	-	-	-	-	-	-	-	-
0.1	3	3	3	3	3	3	0	0	0	0	0	0	100	100	100

* Sensitivity was calculated as the percentage of MALDI-confirmed R5 peptoid beads retained out of the total R5 peptoid beads added. Data from triplicate experiments are shown.
